# Development and initial validation of a new functional ability tool for juvenile dermatomyositis

**DOI:** 10.1186/1546-0096-9-S1-P61

**Published:** 2011-09-14

**Authors:** GC Varnier, A Consolaro, C Malattia, AP Rao, A Madeo, E Demirkaya, D Lazarevic, A Civino, A Martini, A Ravelli

**Affiliations:** 1 Pediatria II, IRCCS G.Gaslini, Genova, Italy; 2 Dipartimento di Pediatria, Università degli Studi di Genova, Italy; 3 Az. Osp. Card.G. Panico, Tricase (LE), Italy

## Background

The assessment of functional ability is an essential part of the clinical evaluation of patients with juvenile dermatomyositis (JDM). However, no physical function tool specific for JDM is currently available.

## Objectives

To develop and validate a new functional ability questionnaire specific for JDM, named Juvenile Myositis Functionality Scale (MyoFun).

## Methods

The MyoFun assesses the ability of the child to perform 15 activities that require the use of all skeletal muscles and muscle groups. Each item is scored from 0 to 3 (0=with no difficulty; 1=with some difficulty; 2=with much difficulty; 3=unable to do). The total score ranges from 0 (normal physical function) to 45 (worst physical function). A parent of 27 children with JDM was asked to complete the MyoFun and the Childhood Health Assessment Questionnaire (C-HAQ) and to rate the child’s overall well-being and pain intensity on a 21-numbered circle visual analog scale (VAS). The attending physician assessed the child’s muscle strength/function with the Kendall’s Manual Muscle Testing (MMT) and the Childhood Myositis Assessment Scale (CMAS). Laboratory tests included creatine phosphokinase (CK) and lactic dehydrogenase (LDH). Construct validity of the MyoFun was examined by calculating its Spearman’s correlations with the other JDM outcome measures on both cross-sectional data and change between 2 consecutive visits. Correlations were considered high, moderate or low when >0.7, 0.4-0.7, or <0.4, respectively

## Results

The MyoFun was found to be feasible, requiring < 5 minutes to be completed and < 2 minutes to be scored. The table shows the Spearman’s correlations of the MyoFun score with the scores of the other JDM outcome measures.

**Table:** 

	MMT	CMAS	C-HAQ	Well-being VAS	Pain VAS	CK	LDH
MyoFun	-0.78	-0.66	0.90	0.85	0.89	0.27*	0.43

A second visit made within 6 months from baseline visit was available for 13 patients. Spearman’s correlation between the change in the MyoFun score and the change in the MMT score was -0.90.

## Conclusion

Preliminary validation analyses have shown that the MyoFun is feasible and has an excellent construct validity and is, thus, suitable for the assessment of functional ability in children with JDM.

